# Biceps strength drop at diagnosis is associated with deltoid strength recovery in severe postoperative C5 palsy: a retrospective cohort study

**DOI:** 10.3389/fsurg.2026.1884729

**Published:** 2026-08-26

**Authors:** Yu-Fei Yuan, Ye Tian, Peng-Fei Shen, Jun Miao

**Affiliations:** 1 Clinical School/College of Orthopedics, Tianjin Medical University, Tianjin, China; 2 Department of Orthopedics, HanDan Center Hospital, HanDan, Hebei, China; 3 Biomedical Engineering, Academic Division of Medicine, Tianjin University, Tianjin, China; 4 Department of Orthpedics, The First Affiliated Hospital of Hebei North University, Zhangjiakou, Hebei, China; 5 Second Department of Orthopedics, Tianjin Jizhou District People’s Hospital, Tianjin, China; 6 Department of Spine Surgery, Tianjin Hospital, Tianjin University, Tianjin, China

**Keywords:** biceps brachii strength recovery, C5 root palsy, cervical decompression, deltoid strength recovery, prediction

## Abstract

**Objective:**

C5 nerve root palsy (C5P) following cervical decompression surgery remains a frustrating complication affecting patients' quality of life. C5P primarily affects the deltoid muscle, with the biceps brachii also involved in more severe cases. This study aimed to investigate the association between the degree of biceps brachii weakness and the recovery of deltoid muscle strength in patients with severe C5P.

**Methods:**

A retrospective cohort study of 78 patients with new-onset severe C5P following unilateral open-door laminoplasty between 2010 and 2024 was analyzed. All patients were retrospectively enrolled and underwent standardized clinical follow-up assessments at 6 and 12 months postoperatively. Univariate and multivariate logistic regression analyses were performed at the 6-month and 12-month time points to investigate the predictive value of patient characteristics, radiological features, and biceps brachii muscle strength for recovery from severe C5P.

**Results:**

A total of 78 patients developed severe C5P after surgery. Univariate logistic regression analysis revealed that biceps brachii strength deficit at diagnosis, high-intensity zone (HIZ) at C3/4 and C4/5, and cervical kyphosis were potential risk factors for lack of deltoid strength recovery at the 6-month follow-up. At the 12-month follow-up, biceps brachii strength deficit at diagnosis, HIZ at C4/5, and cervical kyphosis remained potential predictors of poor recovery of deltoid strength. Multivariate logistic regression identified the degree of biceps brachii strength deficit as an independent predictor of non-recovery at both 6 months (OR = 2.18, *P* < 0.001) and 12 months (Model 1: OR = 3.05, *P* < 0.001; Model 2: OR = 3.10, *P* < 0.001).

**Conclusion:**

The degree of biceps brachii strength deficit is an independent predictor of intermediate- and long-term failure of deltoid muscle recovery following severe postoperative C5P. Identifying this risk factor may provide a critical time reference for timely consideration of nerve transfer surgery in affected patients.

## 1 Introduction

Posterior cervical laminoplasty (commonly referred to as unilateral open-door laminoplasty) is widely used for the treatment of multilevel cervical spondylotic myelopathy (CSM) and ossification of the posterior longitudinal ligament (OPLL) (1, 2). Due to its safety, technical simplicity, and favorable surgical outcomes, this procedure has been extensively adopted by spinal surgeons worldwide (3, 4). However, C5 nerve root palsy remains a debilitating complication following cervical spine surgery—particularly after posterior approaches—with reported incidence rates ranging from 1% to 30% (5–11). This condition typically manifests as delayed-onset motor weakness in the deltoid and biceps brachii muscles and may occasionally be accompanied by dermatomal sensory disturbances, significantly impairing patients' functional recovery and quality of life (12–14).

The incidence of C5P has decreased with improvements in surgical techniques, such as the guttering on the lateral mass technique of laminoplasty (15). However, the exact etiology of postoperative C5P remains unclear and is likely multifactorial. Proposed mechanisms include nerve root traction during spinal cord drift following decompression, and ischemic injury to the spinal cord (16–19). Previous studies have identified various risk factors including demographic characteristics and imaging parameters (7, 20, 21) and iatrogenic injury, which further complicates prognosis (11, 22).

Most patients experience spontaneous recovery within 6–12 months, but approximately 20%–30% of cases result in persistent motor deficits necessitating rehabilitation or surgical intervention (23–26).

The objective of this study was to identify risk factors influencing the time to deltoid strength recovery in patients with severe postoperative C5P. Severe C5P was defined as deltoid weakness (MMT ≤ 3/5) with concurrent biceps brachii weakness (MMT ≤ 3/5). The detailed definition is provided in the Methods section. Understanding the natural recovery trajectory of severe C5P is critical for determining the optimal timing of nerve transfer surgery—particularly in patients with a very low likelihood of spontaneous recovery of deltoid and biceps strength, for whom early surgical intervention may significantly alter functional outcomes.

## 2 Materials and methods

### 2.1 Subjects

A retrospective study was conducted on 7,091 patients with OPLL or CSM who underwent unilateral open-door laminoplasty between January 2010 and December 2024.

Deltoid and biceps strength were assessed by one trained and qualified attending spinal surgeon using manual muscle testing (MMT). C5 palsy (C5P) was defined as a postoperative decline of at least one MMT grade in deltoid and/or biceps strength compared to preoperative status—regardless of whether preoperative strength was normal or already impaired—provided that iatrogenic intraoperative nerve injury was excluded. In our study, recovery of deltoid and biceps strength was defined as return to the patient's preoperative MMT grade. The MMT scale ranges from 0 to 5, with grade 0 indicating no visible muscle contraction and grade 5 representing normal muscle strength. The timing of C5P onset was recorded at the time of first symptom recognition.

Postoperative rehabilitation protocols were standardized across patients. The protocol consisted of three phases: (1) early phase (weeks 1–2): passive range-of-motion exercises and isometric contractions of the deltoid and biceps muscles, initiated within 48 h after surgery; (2) intermediate phase (weeks 3–6): progressive active-assisted and active range-of-motion exercises, with gradual introduction of light resistance training using elastic bands; and (3) late phase (weeks 7–12): strengthening exercises with increasing resistance, functional training for activities of daily living, and neuromuscular re-education. All patients received supervised physical therapy sessions three times per week, with home exercise programs prescribed for non-session days. The rehabilitation protocol was delivered by licensed physical therapists under the supervision of the attending spinal surgeon, with progression criteria based on individual tolerance and pain control (VAS < 4).

The median time from surgery to the diagnosis of C5 palsy was 14 days (range: 3 to 42 days). Among the 78 patients, 68 (87.2%) developed C5 palsy within the first 3 weeks postoperatively, and 10 (12.8%) between 3 and 6 weeks. All patients were assessed at the time of first symptom recognition and subsequently followed up according to a standardized rehabilitation protocol.

Inclusion criteria were: (1) age ≥18 years; (2) primary cervical spine surgery performed via unilateral open-door laminoplasty; (3) Severe C5P was defined as deltoid weakness (MMT ≤ 3/5) with concurrent biceps brachii weakness (MMT ≤ 3/5). (4) complete preoperative and postoperative records; and (5) minimum follow-up duration of 1 year.

Exclusion criteria included: (1) age < 18 years; (2) upper extremity peripheral neuropathy, trauma, tumors, infection; (3) revision surgery; (4) incomplete imaging datasets; (5) loss to follow-up.

Patients were grouped according to whether they developed severe C5P at diagnosis, and further stratified by recovery time (>6 months vs. < 6 months; >12 months vs. < 12 months).

This study was approved by the Ethics Committee of Tianjin Hospital (Approval No. 2025-173). The requirement for informed consent was waived by the Ethics Committee due to the retrospective nature of the study. All patient records and information were fully anonymized before analysis; authors had no access to identifiable participant information during or after data collection. Data were accessed on 15 January 2025 for research purposes.

### 2.2 Demographic variables

Clinical data were obtained from electronic medical records, including age, sex, body mass index (BMI), symptom duration, and Charlson comorbidity index (CCI). Deltoid and biceps strength were recorded at the preoperative visit, at diagnosis of severe C5P, and at 6-month and 12-month postoperative timepoints.

### 2.3 Radiographic variables

Diagnosis (OPLL vs CSM), HIZ segment (C3/4, C4/5, C5/6, C6/7), cervical alignment (lordosis, straight, sigmoid, kyphosis) (27), open-door side (left/right), number of decompressed levels.

### 2.4 Statistical methods

SPSS 25 (IBM, USA) was used for statistical analysis. Continuous variables were presented as $\bar{X}\pm s$ or median with interquartile range depending on data distribution assessed via Shapiro–Wilk test. Categorical variables were presented as count and percentage. Student's *t*-test or Mann–Whitney *U* test was used for continuous variables, and chi-square test or Fisher's exact tests for categorical variables. *P* values <0.05 were considered statistically significant.

Univariate logistic regression was performed to identify potential predictors of deltoid strength recovery at 6 months and 12 months, respectively. Variables significant in univariate analysis (*P* < 0.05) were considered for inclusion in the multivariate logistic regression model, depending on the sample size. In both univariate and multivariate regression analyses, the dependent variable *Y* was defined as the deltoid strength recovery status: *Y* = 1 indicated “non-recovery,” and *Y* = 0 indicated “recovery.” Independent variables included: sex (male=1, female=0), diagnosis (OPLL=1, CSM=0), HIZ (present=1, absent=0), cervical alignment (lordosis=1, straight/sigmoid/kyphosis=0), and open-door side (left=1, right=0). OR > 1 indicated that the factor was associated with a reduced likelihood of recovery.

Given the limited number of outcome events, multivariable models were restricted to a maximum of 2 covariates based on the events-per-variable (EPV) rule of 10 events per predictor. Therefore, we built separate models to evaluate the independent effect of biceps strength deficit after adjusting for each key confounder individually. For the 12-month multivariable analyses (Tables 1, 2), we strictly adhered to the 2-covariate limit due to the smaller number of outcome events (*n* = 23). For the 6-month analysis (Table 3), which had 55 outcome events, we included up to 5 covariates (55/5 = 11 EPV), which also satisfies the 10 EPV rule.

**Table 1:** Multivariable logistic regression- deltoid strength no recovery by 1 year.

Variables	*β*	OR	95%CI	*P*-value
Biceps strength drop at diagnosis	1.12	3.05	2.10–5.25	<0.001
HIZ C4/5	1.05	2.86	1.15–7.10	0.024

**Table 2:** Multivariable logistic regression- deltoid strength no recovery by 1 year.

Variables	*β*	OR	95%CI	*P*-value
Biceps strength drop at diagnosis	1.13	3.10	2.15–5.48	<0.001
Kyphosis	0.90	2.46	1.02–5.93	0.045

CI, confidence interval; OR odds ratio.

**Table 3:** Multivariable logistic regression- deltoid strength no recovery by 6 months.

Variables	*Β*	OR	95%CI	*P*-value
Biceps strength drop at diagnosis	0.78	2.18	1.45–3.28	<0.001
Deltoid MMT grade at diagnosis	−0.52	0.59	0.38–0.92	0.020
HIZ C3/4	1.25	3.49	1.32–9.24	0.012
HIZ C4/5	1.10	3.00	1.18–7.56	0.021
Sigmoid	0.60	1.82	0.70–4.75	0.220
Kyphosis	0.95	2.59	1.05–6.40	0.038

CI, confidence interval; OR, odds ratio.

## 3 Results

A total of 7,091 patients underwent posterior cervical unilateral open-door laminoplasty. Of these, 78 patients (1.1%) met the criteria for severe C5P (Table 4). The median time from surgery to the diagnosis of severe C5P was 14 days (range: 3 to 42 days). Among the 78 patients, 68 (87.2%) developed C5P within the first 3 weeks postoperatively, and 10 (12.8%) between 3 and 6 weeks. Two patients were lost to follow-up before 4 months postoperatively, and four additional patients were lost between 4 and 9 months. Consequently, 76 patients had complete data available for analysis at the 6-month time point, and 72 patients at the 12-month time point. Among them, 21 of 76 patients (27.6%) achieved full recovery at 6 months (Table 5), and 49 of 72 patients (68.1%) achieved full recovery at 12 months (Table 6).

**Table 4:** Baseline characteristics of patients with severe C5 palsy.

Variables	With severe C5 palsy (*N* = 78 )
Age, years	64.41 ± 10.68
Sex	
Male	59 (75.6%)
Female	19 (24.4%)
BMI, kg/m^2^	30.34 ± 5.80
Duration, months	25.58 ± 8.12
CCI	3.27 ± 1.82
Diagnosis	
OPLL	40 (51.3%)
CSM	38 (48.7%)
HIZ segment^†^	
C3/4	30/78 (38.5%)
C4/5	50/78 (64.1%)
C5/6	18/78 (23.1%)
C6/7	6/78 (7.7%)
Cervical alignment	
lordosis	15 (19.2%)
straight	10 (12.8%)
Sigmoid	11 (14.1%)
Kyphosis	42 (53.8%)
Open-door side	
Left	59 (75.6%)
Right	19 (24.4%)
Levels decompressed	4.46 ± 0.66
Pre-op Biceps strength	4.59 ± 0.61
Pre-op Deltoid strength	4.67 ± 0.60
Biceps strength at diagnosis	3.24 ± 1.13
Deltoid MMT grade at diagnosis	2.15 ± 1.13
Biceps strength drop at diagnosis	1.42 ± 1.18
Deltoid strength drop at diagnosis	2.44 ± 1.15

BMI, body mass index; CCI, Charlson comorbidity index; OPLL, ossification of the posterior longitudinal ligament; CSM, cervical spondylotic myelopathy; HIZ, high-intensity zone.

^†^HIZ could present at multiple cervical levels in the same patient; therefore, percentages sum to >100%.

**Table 5:** Baseline characteristics of patients according to 6 months recovery and no recovery of deltoid strength.

Variables	Recovery (*N* = 21 )	No recovery (*N* = 55 )	*P*-value
Age, years	62.86 ± 11.23	65.29 ± 10.57	0.245
Sex			0.300
Male	14 (66.7%)	43 (78.2%)	
Female	7 (33.3%)	12 (21.8%)	
BMI, kg/m^2^	28.93 ± 5.72	30.90 ± 5.85	0.208
Duration, months	24.05 ± 8.66	26.52 ± 7.75	0.260
CCI	2.95 ± 2.11	3.35 ± 1.70	0.429
Diagnosis			0.098
OPLL	14 (66.7%)	25 (45.5%)	
CSM	7 (33.3%)	30 (54.5%)	
HIZ segment			
C3/4	4/21 (19.0%)	25/55 (45.5%)	0.034
C4/5	8/21 (38.1%)	41/55 (74.5%)	0.003
C5/6	3/21 (14.3%)	15/55 (27.3%)	0.174
C6/7	1/21 (4.8%)	5/55 (9.1%)	1.000
Cervical alignment			<0.001
lordosis	12 (57.1%)	2 (3.6%)	
straight	6 (28.6%)	4 (7.3%)	
Sigmoid	2 (9.52%)	8 (14.6%)	
Kyphosis	1 (4.8%)	41 (74.6%)	
Open-door side			0.234
Left	18 (85.7%)	40 (72.7%)	
Right	3 (14.3%)	15 (27.3%)	
Levels decompressed	4.62 ± 0.50	4.42 ± 0.69	0.225
Pre-op Biceps strength	4.86 ± 0.36	4.62 ± 0.65	0.128
Pre-op Deltoid strength	4.90 ± 0.30	4.47 ± 0.66	0.001
Biceps strength at diagnosis	4.05 ± 0.86	2.95 ± 1.10	＜0.001
Deltoid strength at diagnosis	2.95 ± 0.86	1.80 ± 1.04	＜0.001
Biceps strength drop at diagnosis	0.81 ± 0.81	1.67 ± 1.23	0.004
Deltoid strength drop at diagnosis	1.95 ± 0.74	2.67 ± 1.20	0.012

BMI body mass index, CCI Charlson comorbidity index, OPLL Ossification of the Posterior Longitudinal Ligament, CSM Cervical Spondylotic Myelopathy, HIZ High-Intensity Zone.

**Table 6:** Baseline characteristics of patients according to 1 year recovery and no recovery of deltoid strength.

Variables	Recovery (*N* = 49 )	No Recovery (*N* = 23 )	*P*-value
Age, years	62.47 ± 10.88	67.26 ± 9.30	0.073
Sex			0.884
Male	37 (75.5%)	17 (73.9%)	
Female	12 (24.5%)	6 (26.1%)	
BMI, kg/m^2^	30.30 ± 5.88	30.86 ± 6.16	0.717
Duration, months	25.71 ± 7.96	24.84 ± 7.48	0.514
CCI	3.33 ± 1.86	3.13 ± 1.82	0.615
Diagnosis			0.55
OPLL	24 (49.0%)	13 (56.5%)	
CSM	25 (51.0%)	10 (43.5%)	
HIZ segment			
C3/4	15/49 (30.6%)	12/23 (52.2%)	0.078
C4/5	25/49 (51.0%)	22/23 (95.7%)	<0.001
C5/6	11/49 (22.5%)	6/23 (26.1%)	0.735
C6/7	3/49 (6.1%)	3/23 (13.0%)	0.376
Cervical alignment			<0.001
lordosis	14 (28.6%)	0 (0.0%)	
straight	9 (18.4%)	0 (0.0%)	
Sigmoid	9 (18.4%)	1 (4.4%)	
Kyphosis	17 (34.7%)	22 (95.7%)	
Open-door side			0.148
Left	35 (71.4%)	20 (87.00%)	
Right	14 (28.6%)	3 (13.0%)	
Levels decompressed	4.53 ± 0.65	4.39 ± 0.89	0.507
Pre-op Biceps strength	4.59 ± 0.67	4.87 ± 0.34	0.071
Pre-op Deltoid strength	4.63 ± 0.60	4.52 ± 0.67	0.470
Biceps strength at diagnosis	3.63 ± 0.97	2.57 ± 1.12	<0.001
Deltoid strength at diagnosis	2.39 ± 1.00	1.65 ± 1.19	0.010
Biceps strength drop at diagnosis	0.96 ± 0.96	2.30 ± 1.15	<0.001
Deltoid strength drop at diagnosis	2.24 ± 0.92	2.87 ± 1.46	0.030

BMI, body mass index; CCI, Charlson comorbidity index; OPLL, ossification of the posterior longitudinal ligament; CSM, cervical spondylotic myelopathy; HIZ, high-intensity zone.

Univariate logistic regression showed that among patients who did not recover deltoid strength within 6 months postoperatively, the proportions of those with cervical kyphosis, HIZ at C3/4, and HIZ at C4/5 were significantly higher (Table 7).

**Table 7:** Univariate logistic regression- deltoid strength no recovery by 6 months.

Exposure	Statistics	OR (95%CI)	*P*-value
Age, years	64.62 ± 10.74	1.02 (0.97, 1.07)	0.376
Sex			
Male	57 (75.0%)	Ref	
Female	19 (25.0%)	0.56 (0.18, 1.69)	0.303
BMI, kg/m^2^	30.36 ± 5.84	1.07 (0.97, 1.17)	0.190
Duration, months	25.84 ± 8.03	1.04 (0.98, 1.11)	0.232
CCI	3.24 ± 1.82	1.13 (0.85, 1.50)	0.398
Diagnosis			
OPLL	39 (51.3%)	Ref	
CSM	37 (48.7%)	2.40 (0.84, 6.87)	0.103
HIZ C3/4			
absent	47 (61.8%)	Ref	
present	29 (38.2%)	3.54 (1.05, 11.90)	0.041
HIZ C4/5			
absent	27 (35.5%)	Ref	
present	49 (64.5%)	4.76 (1.63, 13.87)	0.004
HIZ C5/6			
absent	58 (76.3%)	Ref	
present	18 (23.7%)	3.90 (0.81, 18.71)	0.089
HIZ C6/7			
absent	70 (92.1%)	Ref	
present	6 (7.9%)	2.00 (0.22, 18.21)	0.539
Cervical alignment			
lordosis	14 (18.4%)	Ref	
straight	10 (13.2%)	4.00 (0.56, 28.40)	0.166
Sigmoid	10 (13.2%)	5.49 (1.78, 26.97)	0.014
Kyphosis	42 (55.3%)	8.50 (2.50, 28.90)	<0.001
Open-door side			
Left	58 (76.3%)	Ref	
Right	18 (23.7%)	2.25 (0.58, 8.76)	0.242
Levels decompressed	4.47 ± 0.64	0.59 (0.25, 1.39)	0.225
Pre-op Biceps strength	4.86 ± 0.59	0.38 (0.11, 1.32)	0.129
Pre-op Deltoid strength	4.59 ± 0.61	0.16 (0.04, 0.69)	0.015
Biceps strength at diagnosis	3.25 ± 1.14	0.31 (0.16, 0.61)	0.0006
Deltoid strength at diagnosis	2.12 ± 1.12	0.30 (0.16, 0.58)	0.0003
Biceps strength drop at diagnosis	1.43 ± 1.19	2.19 (1.12, 3.89)	0.007
Deltoid strength drop at diagnosis	2.47 ± 1.14	1.91 (1.12, 3.23)	0.017

BMI, body mass index; CCI, Charlson comorbidity index; OPLL, ossification of the posterior longitudinal ligament; CSM, cervical spondylotic myelopathy; HIZ, high-intensity zone; Ref*,* reference; CI, confidence interval; OR, odds ratio.

Additionally, deltoid strength recovery at 6 months postoperatively was associated with greater preoperative deltoid and biceps strength, higher deltoid and biceps strength at diagnosis, and a smaller magnitude of strength drop in these muscles from baseline to diagnosis (Table 7). Receiver operating characteristic (ROC) curve analysis was performed to evaluate the predictive performance of biceps strength drop at diagnosis for non-recovery of deltoid strength at 6 months postoperatively. The area under the curve (AUC) was 0.704 (95% CI: 0.582–0.825, *P* = 0.006), indicating moderate discriminative ability. The optimal cutoff value for biceps strength drop at diagnosis was ≥ 1.5 MMT grades, yielding a sensitivity of 78% and a specificity of 74%.

Univariate logistic regression showed that among patients who did not recover deltoid strength by 12 months postoperatively, HIZ at C3/4 and HIZ at C4/5 were more prevalent. In contrast, patients who achieved deltoid strength recovery had higher preoperative deltoid and biceps strength, higher deltoid and biceps strength at diagnosis, and a smaller strength drop at diagnosis (Table 8).

**Table 8:** Univariate logistic regression- deltoid strength no recovery by 1 year.

Exposure	Statistics	OR（95%CI）	*P*-value
Age, years	64.00 ± 10.34	1.05 (0.99, 1.10)	0.078
Sex			
Male	54 (75.0%)	Ref	
Female	18 (25.0%)	1.09 (0.35, 3.39)	0.884
BMI, kg/m^2^	30.48 ± 5.93	1.02 (0.93, 1.10)	0.706
Duration, months	25.43 ± 7.76	0.99 (0.92, 1.05)	0.657
CCI	3.27 ± 1.83	0.87 (0.63, 1.20)	0.399
Diagnosis			
OPLL	37 (51.4%)	Ref	
CSM	35 (48.6%)	0.74 (0.27, 2.00)	0.551
HIZ C3/4			
absent	47 (61.8%)	Ref	
present	27 (37.5%)	2.47 (0.89, 6.85)	0.082
HIZ C4/5			
absent	27 (35.5%)	Ref	
present	47 (65.3%)	5.20 (1.80, 15.00)	0.002
HIZ C5/6			
absent	58 (76.3%)	Ref	
present	17 (23.6%)	1.22 (0.39, 3.84)	0.735
HIZ C6/7			
absent	70 (92.1%)	Ref	
present	6 (8.3%)	2.30 (0.43, 12.39)	0.332
Cervical alignment			
lordosis	14 (19.4%)	Ref	
straight	9 (12.5%)	1.58 (0.19, 13.47)	0.674
Sigmoid	10 (13.9%)	5.63 (1.02, 31.18)	0.053
Kyphosis	39 (54.2%)	6.14 (1.32, 28.46)	0.028
Open-door side			
Left	55 (76.4%)	Ref	
Right	17 (23.6%)	0.37 (0.10, 1.46)	0.158
Levels decompressed	4.49 ± 0.73	0.82 (0.42, 1.62)	0.571
Pre-op Biceps strength	4.68 ± 0.58	0.33 (0.10, 1.15)	0.081
Pre-op Deltoid strength	4.59 ± 0.62	0.75 (0.34, 1.65)	0.478
Biceps strength at diagnosis	3.29 ± 1.01	0.37 (0.21, 0.66)	0.0008
Deltoid strength at diagnosis	2.15 ± 1.06	0.52 (0.31, 0.86)	0.012
Biceps strength drop at diagnosis	1.39 ± 1.02	3.14 (1.76, 5.59)	0.0001
Deltoid strength drop at diagnosis	2.44 ± 1.11	1.64 (1.03, 2.59)	0.036

Ref*,* reference; CI, confidence interval; OR, odds ratio.

Multivariable logistic regression showed that a larger biceps strength drop at diagnosis, HIZ at C3/4, HIZ at C4/5, and kyphosis were independent risk factors for lack of deltoid strength recovery at 6 months (OR = 2.18, OR = 3.49, OR = 3.00, and OR = 2.59, respectively). After adjusting for initial deltoid MMT grade at diagnosis, the association between biceps strength drop and poor deltoid recovery remained significant (OR = 1.45, 95%CI: 1.18–1.78, *P* = 0.001) (Table 3). A larger biceps strength drop at diagnosis, HIZ at C4/5, and kyphosis were independent risk factors for lack of deltoid strength recovery at 12 months (OR = 3.05, OR = 3.10, and OR = 2.59, respectively) (Tables 1, 2).

Among patients who had not fully recovered by 12 months, 11 out of 23 achieved functional recovery (MMT ≥ 3) at last follow-up, while 12 out of 23 showed minimal improvement.

## 4 Discussion

Postoperative C5P remains a troubling complication. This retrospective cohort study identified biceps muscle strength deficit at diagnosis as an independent predictor of deltoid strength recovery at 6 and 12 months in patients with severe postoperative C5P. Severe C5P concurrently affects both the deltoid and biceps brachii muscles, and the time required for strength recovery in these muscles directly influences patients' ability to return to daily life and work, as well as their overall quality of life. Simultaneous involvement of both muscles significantly impairs essential activities of daily living, such as personal hygiene, dressing, and driving (28).

The incidence of severe C5P in our cohort was 1.1%, consistent with previous studies and meta-analyses (8, 10, 14, 17, 28, 29). At 6 months postoperatively, the combined prevalence of HIZ at C3/4 and C4/5 in the non-recovery group exceeded 100%, indicating that some patients had high-intensity signals at both levels simultaneously. In univariate analysis, HIZ at C3/4 and HIZ at C4/5 demonstrated stronger associations with poor prognosis compared to HIZ at other levels (OR = 3.54, *P* = 0.041 and OR = 4.76, *P* = 0.004, respectively), suggesting that concurrent high signal intensity at C3/4 and C4/5 significantly worsens outcomes in severe C5P. This finding aligns with prior research (9).

Furthermore, among non-recovering patients, sigmoid and kyphosis alignment accounted for substantial proportions of cervical sagittal malalignment (14.6% and 74.6%, respectively). Both sigmoid and kyphosis were strongly associated with unfavorable recovery (OR = 5.49, *P* = 0.014 and OR = 8.50, *P* < 0.001, respectively), indicating that these sagittal profile abnormalities hinder deltoid strength recovery in severe C5P. This conclusion is consistent with the findings of Kota and Kazuhiro (27, 30).

However, at 12 months postoperatively, univariate logistic regression showed that sigmoid cervical alignment was not a potential risk factor, whereas HIZ C4/5, kyphosis, and biceps strength deficit at diagnosis remained potential risk factors—with biceps strength deficit at diagnosis showing the most significant P value (P = 0.001)—indicating that biceps strength deficit at diagnosis has a substantial impact on both intermediate- and long-term recovery in severe C5P.

Multivariable logistic regression at 6 months postoperatively showed that, after adjusting for confounding effects of HIZ and cervical alignment, biceps strength deficit at diagnosis remained an independent risk predictor for lack of deltoid strength recovery. At 12 months, multivariable logistic regression further demonstrated that, after separately adjusting for HIZ C4/5 and kyphosis, biceps strength deficit at diagnosis remained the strongest independent predictor of non-recovery of deltoid strength. The OR at 6 months (OR = 2.18) was lower than that at 12 months (OR = 3.05 and OR = 3.10), indicating that biceps strength deficit at diagnosis significantly influences both intermediate- and long-term deltoid recovery.

This finding suggests that a greater biceps strength deficit at diagnosis reflects more severe C5 root injury and portends a very low likelihood of spontaneous recovery. Patients with a greater biceps strength deficit at diagnosis may therefore require closer clinical follow-up and earlier referral to peripheral nerve specialists. However, prospective studies are needed before this parameter can be used to determine the optimal timing of nerve transfer surgery (25, 26).

In both the 6-month and 12-month multivariable logistic regression models, kyphosis was included as a covariate, with OR of 2.59 and 2.46, respectively, indicating that cervical kyphosis adversely affects both early and long-term prognosis (31). This observation also partially supports the “bowstring theory” (30).

Additionally, HIZ C4/5 showed strong predictive value in both models, with OR of 3.00 at 6 months and 2.86 at 12 months, confirming its substantial negative impact on deltoid recovery.

Kunihiko et al. (32) reported intraoperative findings of C5 nerve root “flattening” and other pathological changes in patients who developed postoperative C5P, and hypothesized the presence of subclinical dorsal cord lesions. Such dorsal cord pathology may involve the motor segments of C5 and C6 (33, 34), leading to weakness in both the deltoid and biceps brachii. Notably, Kunihiko et al. (32) proposed that preoperative electromyography (EMG) could be used in future studies to predict the incidence of postoperative C5P. Supporting this, Zheng et al. (35) found in their preoperative EMG study that patients who later developed C5P already exhibited evidence of axonal loss in the C5 nerve root, either ongoing or persisting for several weeks prior to surgery.

Given the differing incidence of C5P between anterior and posterior cervical surgeries, and to minimize outcome variability and reduce inclusion bias, we precisely defined our study population by including only patients who underwent posterior cervical unilateral open-door laminoplasty.

In building the multivariable logistic regression models, we adhered to the widely accepted “10 EPV” rule (36). Accordingly, we limited the number of predictor variables to two and constructed two separate models to mitigate the risk of over-fitting. Based on the results of univariate regression analyses—and guided intuitively by both statistical significance and clinical relevance—we selected HIZ C4/5 and kyphosis as the two predictors for inclusion in the multivariable logistic regression models.

After excluding variables that were non-significant in bivariate analysis, multivariable logistic regression revealed that biceps motor drop at diagnosis was a strong predictor of deltoid weakness in severe C5 palsy at both time points.

As outlined in the Introduction, patients with severe C5P involving concurrent deltoid and biceps brachii weakness experience substantially impaired quality of life, underscoring the clinical need for accurate prediction of recovery trajectory to inform surgical decision-making.

### 5.1 Limitations

This study has several limitations, including those inherent to its retrospective design. First, follow-up assessments were fixed at 6 and 12 months without more granular intervals, which may have obscured the detailed trajectory of recovery. Future studies could employ survival analysis based on time-to-event data to better capture the dynamic prognosis of patients over time.

Second, postoperative confounders were not fully controlled. For example, acute postoperative pain may have interfered with manual muscle testing, and psychological factors in patients could have influenced strength assessments at different postoperative time points.

Third, loss to follow-up may have introduced bias into the results. Patients were lost for various reasons—some may have experienced symptom improvement, while others may have sought care at different medical centers due to worsening symptoms. Regardless of the underlying reason, any loss to follow-up has the potential to bias the study outcomes.

Fourth, this study is a single-center retrospective analysis, which limits the generalizability of the findings. Future prospective, multicenter studies with larger sample sizes are needed to minimize heterogeneity and improve generalizability.

**Figure 1:**
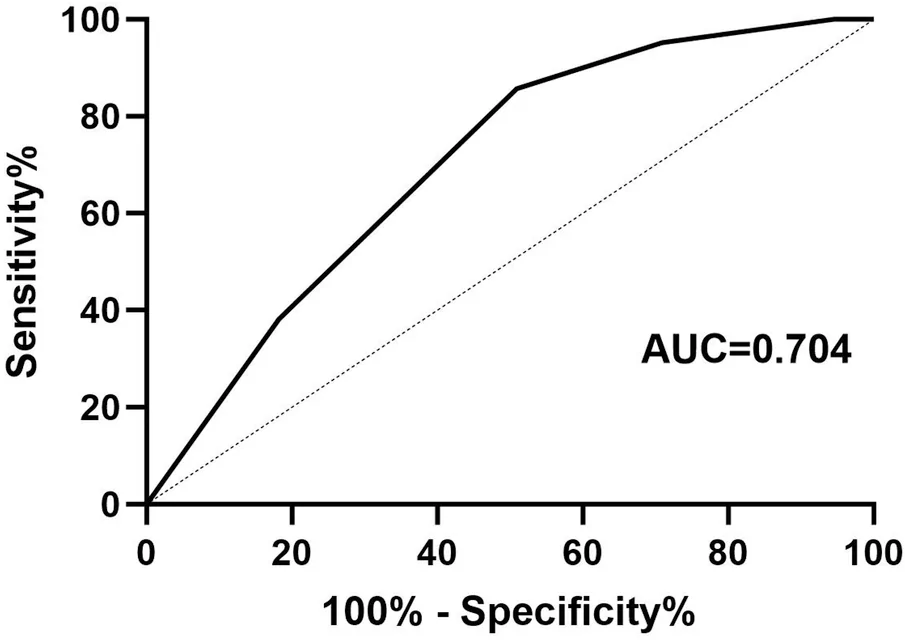
Receiver operating characteristic (ROC) curve of biceps strength drop at diagnosis for predicting non-recovery of deltoid strength at 6 months postoperatively. The area under the curve (AUC) was 0.704 (95% CI: 0.582–0.825, *P* = 0.006). The optimal cutoff value was ≥ 1.5 MMT grades (sensitivity: 78%; specificity: 74%).

Additionally, we acknowledge that biceps brachii weakness at diagnosis may reflect more extensive C5/C6 nerve root involvement rather than serving as a truly independent prognostic factor. Although we adjusted for initial deltoid MMT grade in the multivariable model, residual confounding from unmeasured factors (e.g., preoperative subclinical nerve root injury, individual variability in pain perception) cannot be entirely excluded. Future studies with electrophysiological assessments may help further delineate the independent contribution of biceps strength deficit to recovery outcomes (Figure 1).

## 6 Conclusion

C5P remains a complex and clinically challenging complication following cervical spine surgery, causing significant distress for both patients and clinicians. However, its underlying pathophysiology is still not fully understood. The deltoid muscle—the primary muscle affected in C5P—is critical for upper limb function, and its impairment severely disrupts activities of daily living and work capacity; thus, accurately predicting recovery time is of paramount importance. When both the deltoid and biceps brachii are concurrently involved, functional outcomes are even worse.

This study provides novel evidence regarding the predictive value of biceps strength deficit at diagnosis for deltoid strength recovery following severe postoperative C5P. Biceps strength deficit at diagnosis emerged as the strongest independent predictor of recovery duration in severe C5P. This finding may help identify patients who require closer monitoring and earlier referral to peripheral nerve specialists, though further prospective studies are needed to validate its utility in guiding the timing of nerve transfer surgery.

## Data Availability

The original contributions presented in the study are included in the article/Supplementary Material, further inquiries can be directed to the corresponding author.
